# Alternative splicing complexity contributes to genetic improvement of drought resistance in the rice maintainer HuHan2B

**DOI:** 10.1038/s41598-017-12020-3

**Published:** 2017-09-15

**Authors:** Haibin Wei, Qiaojun Lou, Kai Xu, Ming Yan, Hui Xia, Xiaosong Ma, Xinqiao Yu, Lijun Luo

**Affiliations:** Shanghai Agrobiological Gene Center, Shanghai, 201106 China

## Abstract

Water-saving and drought-resistantce rice (WDR) breeding practices have greatly increased grain yield and drought resistance. To study the genetic basis of adaptation to drought, transcriptome sequences from the WDR maintainer line HuHan2B and the recurrent parent HanFengB were analyzed for alternative splicing (AS) complexity. Intron retention, the dominant AS type, accounted for 42% of the observed AS events. Differential expression analysis revealed transcripts were preferentially expressed in different varieties and conditions. Based on gene ontology predictions, the biological functions of drought-induced transcripts were significantly enriched in genes involved in transcription regulation, chloroplast components and response to abiotic stimulus in HuHan2B, whereas developmental processes for reproduction were primarily enriched in HanFengB. The regulatory network of transcription factors was driven by cohorts of transcript splicing targets, resulting in more diversified regulatory relationships due to AS complexity than in our previous findings. Moreover, several genes were validated to accumulate novel splicing transcripts in a drought-induced manner. Together, these results suggest that HuHan2B and HanFengB share similar AS features but that a subset of genes with increased levels of AS involved in transcription regulatory networks may contribute an additional level of control for genetic improvement of drought resistance in rice maintainer HuHan2B through breeding.

## Introduction

Drought is one of the most severe environmental factors of agricultural limitations, and affects vast regions for cereal crop growth and productivity, and has impact on global food security^1,2^. For a new hope of food security and sustainable agriculture, we have been developing water-saving and drought-resistance rice (WDR) breeding program over a decade^3–5^. The sowing and cultivation of this rice requires only half water of paddy rice, lower inputs and less energy, which makes the WDR very environmentally friendly and economically responsible^3^. However the genetic and molecular mechanisms of enhanced drought resistance of such a WDR are little known.

During the long period of crop domestication and classical plant breeding, plants have evolved a series of mechanisms to acclimate to severe water deficit^6–9^. The negative effects of drought stress are involved in various plant processes, including growth, physiology, metabolism and transcription^10,11^. During post-transcriptional processing, alternative splicing (AS) produces different mature mRNAs and provides a versatile means of genetic regulation and, hence, has important biological consequences for plant growth, development and stress responses^12,13^. Advances in high-throughput technology are enabling a global analysis of AS to dissect its functional characteristics in stress responses^14^. Accumulation of alternatively spliced mRNAs in many studies has revealed the prevalence of AS in plants, including *Arabidopsis thaliana* ^15^, maize (*Zea mays*)^16,17^, cotton (*Gossypium raimondii*)^18^, soybean (*Glycine max*)^19^, pineapple (*Ananas comosus*)^20^, and rice (*Oryza sativa*)^21^. These studies have demonstrated that, among plant AS events, in contrast to animals, intron retention (IR) is the dominant type and has been shown not to be a by-product of incomplete splicing^22^. Increased levels of specifically spliced transcripts can have beneficial effects for development and stress acclimation^23^. AS analysis in maize revealed that drought stress induced large developmental splicing changes in the leaf and ear but relatively few changes in the tassel^17^. Moreover, it is likely that stage-dependent AS events are crucially important, dramatically affecting grain yield^24^. Additionally, during domestication of maize, many genes have been selected for increased AS complexity^25^. Although little is known about the extent and significance of drought-responsive splicing in rice, alternative splicing may provide an explanation for genetic improvement with respect to how drought can impact the offspring’s behavior in WDR breeding.

The increasing scarcity of fresh water for crop production makes the selection of water-saving and drought-resistant rice crucial^2,11^. Genetic materials from the parents are useful for studying gene actions involved in drought response in offspring, such as a new japonica maintainer HuHan2B from WDR breeding^26^. HuHan2B was developed from a backcross breeding procedure that introduced drought resistance into the breeding parent HanFengB (normal lowland rice). Its agronomic traits were characterized in previous studies, and the source of genetic variation was involved in a clear breeding pedigree^3^. In a previous study, the differentially expressed genes between HuHan2B and its recurrent parent HanFengB under drought stress showed that specific inherited alleles were primarily enriched in the regulatory network of transcription factors (TFs) and target genes^26^. However, AS of many genes resulted in the creation of multiple functional mRNAs from pre-mRNAs, and splicing isoforms were specifically expressed in various tissues, which provided a specific mechanism for genetic regulation in plants exposed to environmental stress^23^. The molecular mechanisms of enhanced drought resistance in HuHan2B might be related to increased transcriptome diversity due to genes with increased levels of AS.

In this study, we investigated the complexity of AS in leaf tissue in the offspring HuHan2B and its recurrent parent HanFengB under well-watered (WW) and drought stress (DS) conditions. Transcriptome libraries of the two rice varieties were constructed from developing leaves at three time points during the reproductive stage. This analysis identified novel genes and transcript isoforms from AS events via reference-based transcriptome assembly. The differentially expressed transcripts (DETs) were also analyzed and functionally characterized to uncover the molecular responses of rice to water deficit. The TF and regulatory networks will serve as useful resources for establishing the functional roles of AS events during the drought-stress response in rice. Together, these results highlight the importance of AS with regulatory roles to contribute an additional level of control for drought-responsive genes. Furthermore, our analysis provided some molecular evidence to understand the genetic mechanisms of enhanced drought resistance in WDR breeding by a comprehensive survey of AS at the reproductive stage and will facilitate further genomic and genetic studies.

## Results

### Transcriptome sequencing and reference-based assembly

We previously established a new japonica maintainer, HuHan2B, to study genetic variation and enhanced drought resistance^3,26^. To analyze isoform-level mRNA abundances and AS patterns that were altered during backcross breeding, we performed high-throughput RNA sequencing (RNA-Seq) on the leaves of the offspring HuHan2B and the parent HanFengB at three time points under WW and DS conditions. In total, we obtained more than 110 million raw reads for each rice variety and an average of 57 million reads for each condition (Table 1). These reads were extended to super-reads with an average length of 210 bp using the MaSuRCA assembler. The super-reads contained an average of 35 million maximal pairwise overlap reads for each sample, equivalent to 12× coverage of the genome. Reference-based transcriptome assembly of assembled super-reads and two unassembled paired reads was performed using a TopHat-StringTie pipeline, employing the Nipponbare genome as a reference. The analysis yielded approximately 86.9 to 87.2% overall alignment rates for control and drought samples (Table 1). Over 98% of these reads were unique and mapped only once to a genomic locus. Global inspection of assembled transcripts revealed that a total of 108,946 transcripts were identified (derived from 34,589 unique loci), including 79,575 and 80,259 transcripts (30,614 and 31,095 unique loci) in HuHan2B and HanFengB, respectively (Table 1). This number of assembled transcripts was significantly higher than the 66,153 rice transcripts in the annotated set (MSU7.0), whereas there were fewer detected genes than in the rice annotation set, possibly due to AS events generating isoform-level mRNA abundance during development and drought stress in rice. A summary of the assembly statistics and their corresponding transcripts is provided in Table S1.

**Table 1:** Summary of RNA-Seq reads mapping and transcriptomes based on reference assemblies.

	Raw reads		Super-reads		Mapping rate (%)		Assembled transcripts	
	Control	Drought	Control	Drought	Control	Drought	Total	Novel(loci)
HuHan2B	58,748,817	56,094,897	35,929,209	34,483,019	87.2	87.1	79,575	1,556(1,069)
HanFengB	59,926,181	51,534,977	37,191,409	31,973,630	86.9	87.0	80,259	1,580(1,092)

### Estimation of transcript expression in leaf transcriptomes

The expression levels of known and novel transcripts were estimated by applying StringTie to short read alignment results. The isoform-level expression was normalized to fragments per kilobase of transcript per million mapped fragments (FPKM) in the StringTie process. Because some transcripts were expressed with very low FPKM values, representing low expression or background, we filtered out these transcripts as described in the Methods to obtain a set of 37,403 high-confidence transcripts in the Gffcompare process. The filtered transcript expression was then used for further comparisons among different conditions and two varieties.

**Figure 1:**
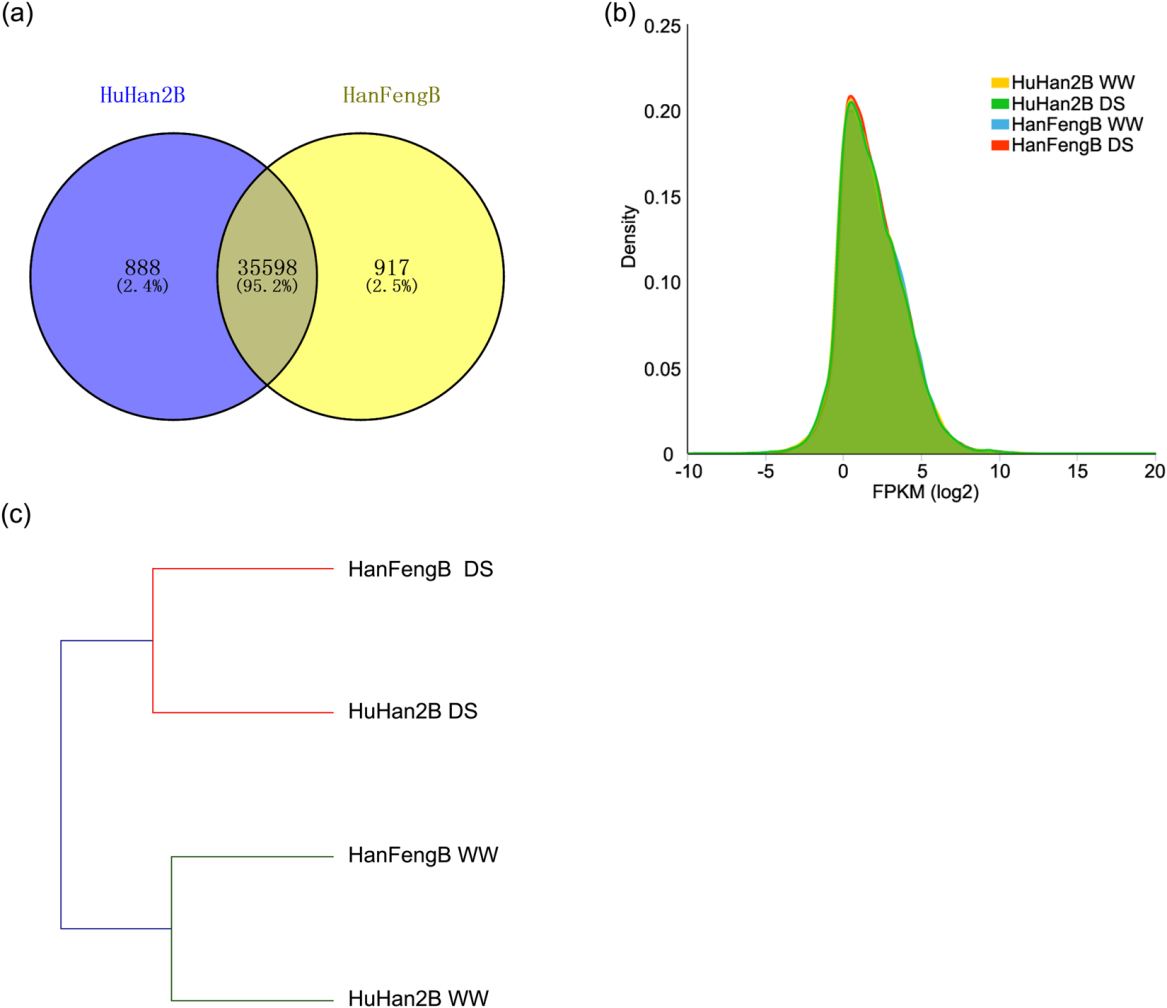
An overview of reference-based transcriptome assembly. (**a**) Detection of overlapped transcript isoforms between the offspring HuHan2B and the recurrent parent HanFengB. (**b**) The probability density distribution of expression in each variety and condition based on the log_2_ FPKM, showing the overall similarity in expression profiles between HuHan2B and HanFengB under each condition. (**c**) Hierarchical clustering indicated that the same treatments were grouped more closely than the same variety.

To obtain an overview of leaf transcriptomes at the reproductive stage, we examined the distribution of expressed transcripts between the offspring HuHan2B and the parent HanFengB. A large number of transcripts (at 95.2%) overlapped in HuHan2B and HanFengB, which showed similar transcriptomes in the offspring and breeding parent (Fig. 1a). We analyzed the density distribution of transcript expression, which hinted that HuHan2B and HanFengB respond to drought stress in a similar manner (Fig. 1b). Clustering analysis showed that the relationships were closer in the same condition than in the same variety (Fig. 1c). Although the overall gene expression levels of the breeding parent and offspring were similar in the above analysis, we investigated divergent drought-responsive regulation through novel transcripts and various AS events in multi-exon genes.

### Identification of novel transcripts and alternative splicing events

**Figure 2:**
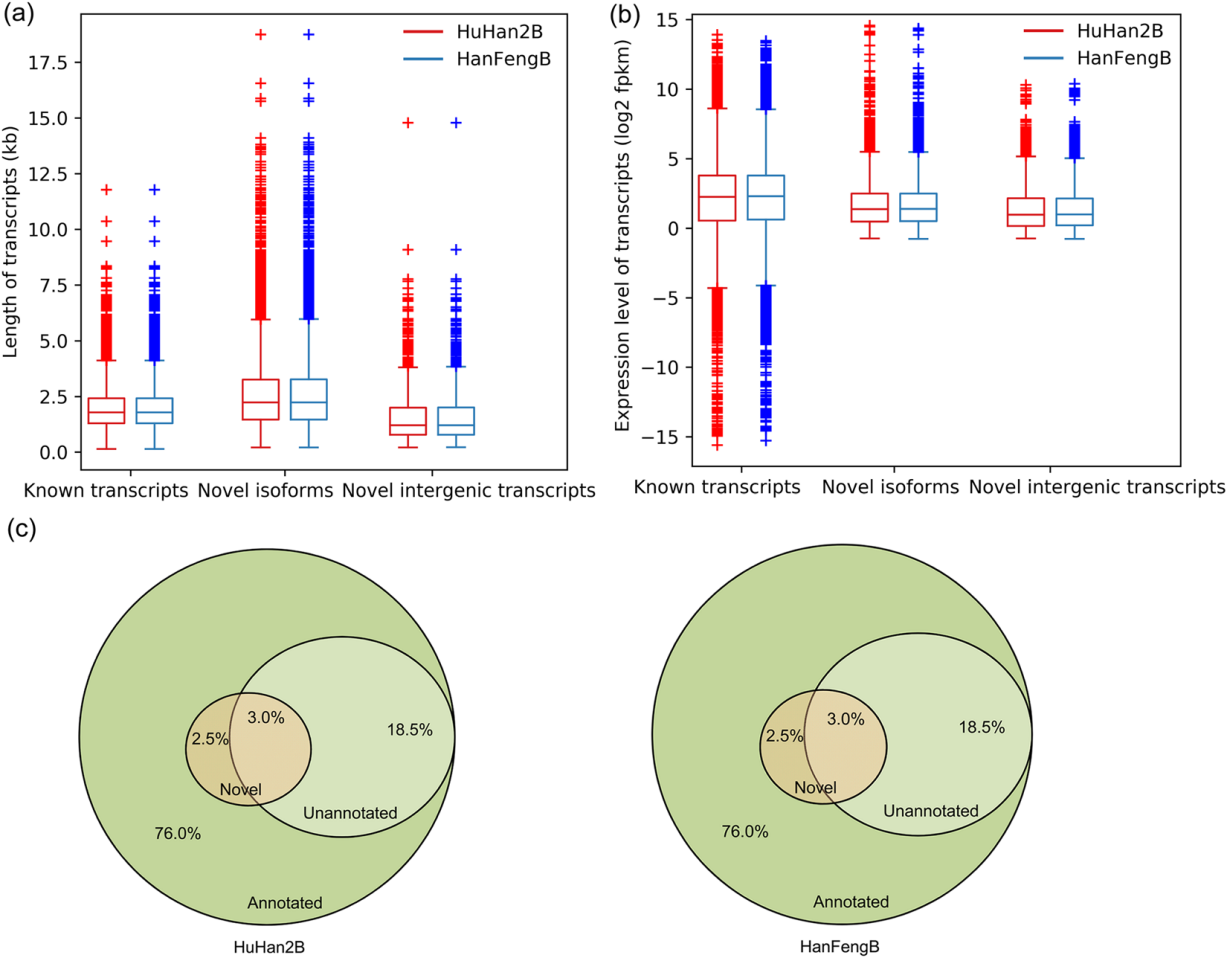
Identification and annotation of assembled transcript isoforms. (**a**) and (**b**) Estimated distributions of length and expression level of transcripts for HuHan2B (red) and HanFengB (blue). These assembled transcripts could be further classified into three categories: known transcripts (left), novel transcript isoforms (center) and novel intergenic transcripts (right, located outside of the annotated gene regions). (**c**) The proportions of the categorized transcripts with respect to annotated, un-annotated, and novel intergenic transcripts (annotated and un-annotated) in HuHan2B and HanFengB rice varieties.

RNA-Seq facilitates the identification of novel genes and transcript isoforms. Of the 36,486 and 36,515 high-confidence transcripts in the offspring HuHan2B and the breeding parent HanFengB, we identified 14,955 and 14,968 novel transcript isoforms, respectively. When compared with known (annotated) transcripts, the average length and length range of novel transcript isoforms were longer (2,524 bp in HuHan2B and 2,532 bp in HanFengB) for both rice varieties (Fig. 2a). In addition, 1,999 (1,408 loci) and 2,015 (1,422 loci) novel intergenic transcripts were also identified in HuHan2B and HanFengB, respectively, whereas the average lengths were shorter (1,533 bp in HuHan2B and 1,532 bp in HanFengB) than known (annotated) transcripts for both rice varieties (Fig. 2a). Box plots of the isoform-level expression (log_2_FPKM) revealed that the average fpkm and fpkm ranges of novel transcript isoforms and intergenic transcripts were less than those of known transcripts in both varieties (Fig. 2b). After functional annotation, more than 70% of the assembled transcripts in both rice varieties were assigned putative functions (Fig. 2c). Surprisingly, more than 45% of novel intergenic transcripts (2.5% of total transcripts) were also assigned putative functions for each rice variety (Fig. 2c). Gene Ontology (GO) term analysis showed that these new transcripts were involved in various biological processes, such as RNA processing/splicing, response to stimulus/stress, regulation of gene expression/transcription, cell cycle processes, signal transmission/transduction, transport, and defense responses, which were closely related to plant growth and abiotic stress responses. In our analysis, leaf transcriptome profiles showed little diversity in terms of the number and distribution of transcripts between HuHan2B and HanFengB, but new transcripts were functionally correlated with RNA processes and stress responses.

**Figure 3:**
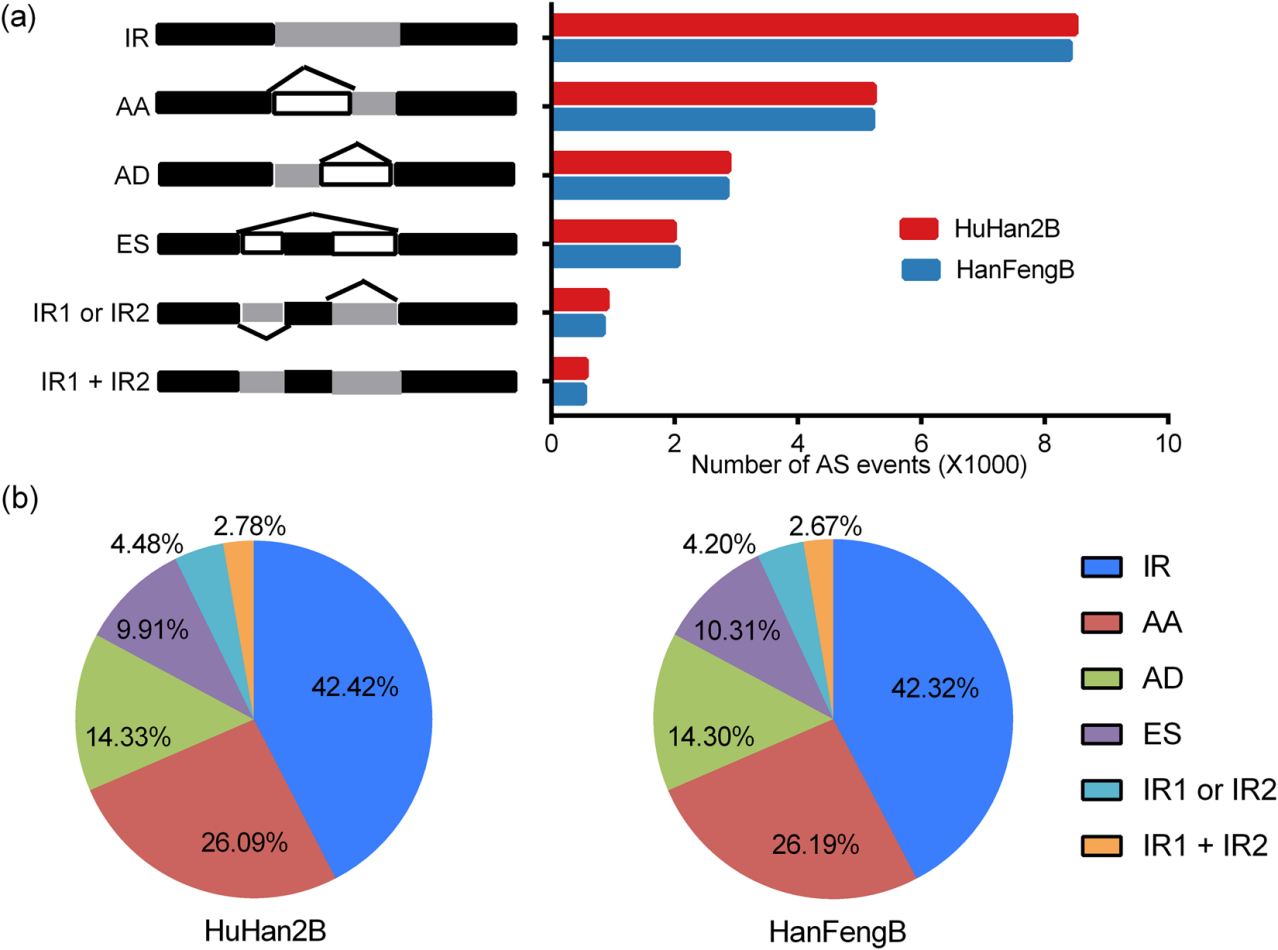
Categories and differences of dominant alternative splicing (AS) events in the transcriptomes of the HuHan2B and HanFengB rice varieties. (**a**) The six splicing types are schematically illustrated, and the numbers of AS events in HuHan2B (red) and HanFengB (blue) are shown. Black indicates annotated exons, white boxes represent introns, and gray boxes denote novel transcript elements. (**b**) Comparison of the proportions of AS events in HuHan2B (left part of the subplot) and HanFengB (right part of the subplot), reflecting a high level of similarity between the AS events in these rice varieties. IR, intron retention; AA, alternate 3′ acceptor; AD, alternate 5′ donor; ES, exon skipping.

To investigate the patterns of alternative splicing of leaf transcriptomes at the reproductive stage, we categorized all alternative transcripts into different types of AS events from assembled transcripts using the AStalavista tool. In this study, we focused on the major AS events with high frequency, including four single-types: IR, alternate 3′ acceptor (AA), alternate 5′ donor (AD), exon skipping (ES), and two mixed-types: IR1 or IR2, IR1 + IR2 (Fig. 3). Altogether, the landscape of different AS groups revealed that IR, AA, AD and ES were the most common types of AS events, as has been shown in previous studies in Arabidopsis, Zea mays and Oryza sativa^15,17,21^. Figure 3 shows that IR was the most frequent AS event, represented by 8,437 and 8,459 events in HuHan2B and HanFengB (Fig. 3a), respectively, accounting for approximately 40% of the AS events (Fig. 3b), which was similar to values reported in previous studies^21^. Moreover, AA and AD events accounted for ~26% and ~14% of the total AS events, respectively, while ES events were the least frequent of the four single-types (Fig. 3b). Among the mixed-type AS events, the IR1 or IR2 events were more frequent, accounting for ~5% in both rice varieties (Fig. 3b). In total, ~32% of multi-exon genes among the high-confidence transcripts underwent AS during the reproductive stage. Overall, the frequency distributions of AS events were very similar between the offspring HuHan2B and the breeding parent HanFengB. AS events can affect the functions of protein coding sequences or generate regulatory mRNAs to control transcript levels; therefore, some AS events may be involved in the differential expression of transcripts.

### Differential transcript expression in comparison to HanFengB WW

**Figure 4:**
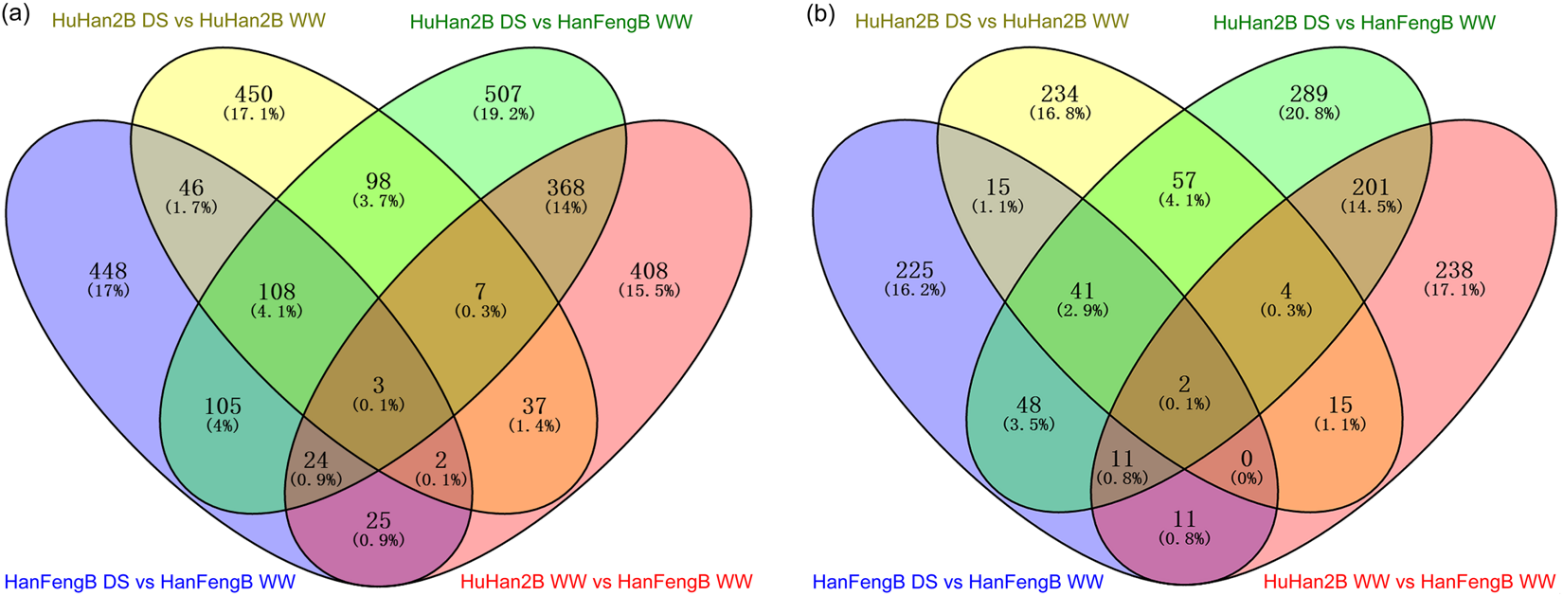
Venn diagrams of the differentially expressed transcripts (DETs) derived from pairwise comparisons between HuHan2B and HanFengB under drought stress. (**a**) The DETs with HanFengB WW was relatively clear. Ratios of DETs arising from the different comparisons were relatively high. (**b**) Half of the overall DETs had splicing events. Treatments: DS, drought stress; WW, well-watered.

RNA-Seq is generally used to determine which genes are up- or down-regulated by comparing gene expression levels between conditions, such as drought stress treatment vs non-treated. We used Ballgown to identify DETs between each treatment and variety from the expression estimates computed in the TopHat-StringTie pipeline. The breeding parent HanFengB under the WW condition was taken as the control/baseline, meeting drought-resistance criteria for drought treatment. Differential expression analysis was used to create 4-way Venn diagrams of the overlap in significant DETs resulting from the comparison to baseline HanFengB WW (Fig. 4a). There were many DETs identified in the various comparisons. Compared with HanFengB WW, relatively large numbers of transcripts were differentially expressed between HuHan2B WW (874) and HuHan2B DS (1,220). More than 500 transcripts were also drought-responsive DETs in both rice varieties under drought stress (Fig. 4a). Most notable was the larger transcriptional differences of drought-responsive DETs between HuHan2B (751 DETs) and HanFengB (761 DETs), as only 159 commonly regulated DETs were shared by both. Although similar numbers of DETs were observed for each rice variety under drought stress, the small proportion of common DETs (~20%) suggested a greater extent of transcriptional variation from the breeding parent HanFengB to the offspring HuHan2B. These differences were likely due to divergent drought response regulatory mechanisms based on novel transcripts and various AS events.

Although the proportion of common DETs between HuHan2B and HanFengB was less, there was a large overlap among the significantly enriched functional categories of DETs under well-watered and drought stress conditions. The shared GO terms in response to abiotic stimulus, water, heat, oxidative stress, osmotic stress, carbohydrate catabolic process, plasmodesma, cell wall and cell junction were overrepresented among GO enrichment categories of DETs in both rice varieties. Certain overrepresented GO terms, such as regulation of transcription, macromolecule biosynthetic process, RNA biosynthetic process, carbohydrate metabolic process, transcription factor complex, chloroplast stroma, response to hormone, salicylic acids, and ethylene, were specific to HuHan2B. In contrast, the overrepresented GO terms specifically enriched in HanFengB included developmental process involved in reproduction, external encapsulating structure, vacuolar membrane, thylakoid, organ development, root development, flower development, response to lipids, and abscisic acid. In contrast, under well-watered conditions, the enriched GO terms of DETs between HuHan2B and HanFengB were involved in inorganic ion transmembrane transport, thylakoid, thylakoid membrane, mitochondrial membrane, sulfate transport, response to nutrient levels, response to starvation, among others. Moreover, the overrepresented GO terms of DETs between HuHan2B DS and HanFengB WW were dominated by activities related to response to abiotic stimulus, osmotic stress, heat, water, cell wall, cell communication, and others. The GO enrichment analysis of DETs suggested that the fundamental distinctions of potentially important GO terms between HuHan2B and HanFengB mainly included two aspects: cellular components of chloroplasts and regulation of transcription. With these distinctions, we could also see that developmental processes involved in reproduction were not affected by drought stress in HuHan2B, based on the difference in overrepresented genes related to the responses to nutrient levels and cellular components of chloroplasts under well-watered conditions. Obviously, different strategies to control transcript levels were adapted to respond to drought stress to protect the developmental process in reproductive stages.

### Differential expression of transcripts with alternative splicing events

To evaluate the effects of drought-induced AS on cellular processes, DETs with six AS types were extracted from the output of the ASTALAVISTA analysis of total assembled transcripts for each rice variety. Approximately half of the DETs were generated as a result of AS events in the rice varieties under drought stress (Fig. 4b). Compared the numbers of DETs with alternative splicing events between HanFengB DS and HanFengB WW, we found that 310 AS transcripts from 281 genes were only differentially expressed between HuHan2B DS and HuHan2B WW (Table S2). Moreover, a similar proportional distribution of the six types of AS events was observed in 310 AS transcripts, and IR was the major AS event in respond to drought stress, accounting for 36% of total AS events from 310 transcripts in Table S2. A few genes showed different AS pattern between HuHan 2B and HanfengB (Table S2). Thus, the effects of AS were appeared in both different AS pattern and differential expression of transcripts.

**Figure 5:**
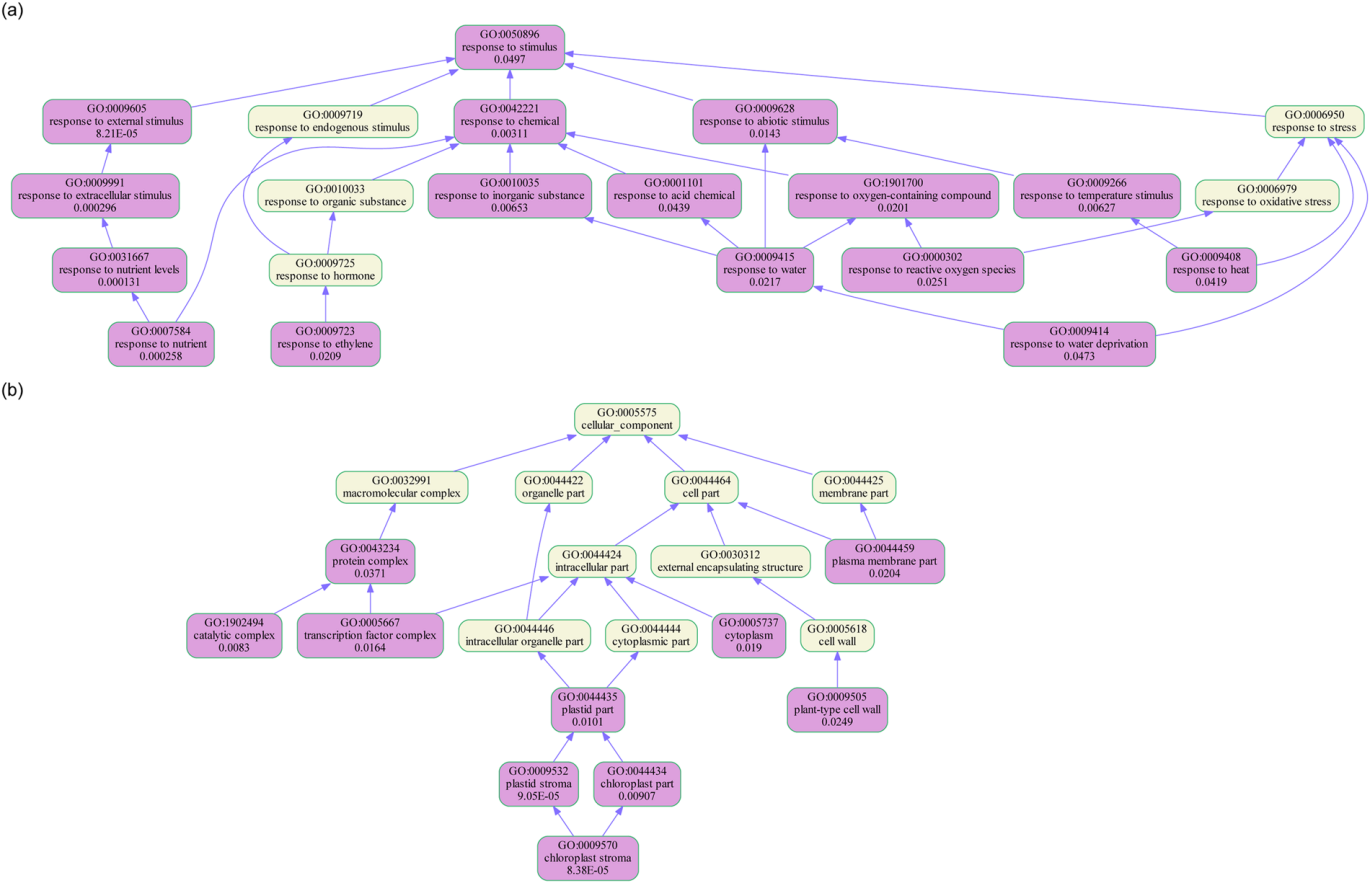
The representative biological processes in GO enrichment of DETs with AS events. (**a**) The responses of functional categories to stimuli were frequently affected by AS events under drought stress. (**b**) Chloroplast processes involved in cellular components were related to energy metabolism of photosynthesis and potential stress responses. Pink indicates significantly overrepresented GO terms.

The functional categorization of DETs with alternative splicing events was similar to that of total DETs. GO classification showed that response to abiotic stimulus, oxygen-containing compound, inorganic substance, cell death, and cell wall terms were significantly enriched under drought stress in both rice varieties. However, GO terms related to response to chemicals, ethylene, regulation of transcription, transcription factor complex, and chloroplasts were significantly enriched in HuHan2B (Fig. 5). GO terms related to response to oxidative stress, developmental process involved in reproduction, organ development, shoot system development, and root development were significantly enriched in HanFengB. Furthermore, the enriched GO terms for splicing isoforms in DETs between HuHan2B and HanFengB under well-watered conditions were related to vacuole, inorganic ion transmembrane transport, response to starvation, nutrient levels, and other factors. The observation that the functional categorization of splicing isoforms was similar to that of total DETs suggested that stress-related gene ontology categories were over-represented in the genes subjected to AS in both rice varieties. The specifically overrepresented GO terms regulation of transcription and chloroplasts were significantly enriched in HuHan2B. We suppose that the enhanced function of regulation of transcription regulated the response to abiotic stimuli and nutrient levels so that the chloroplasts of HuHan2B had well-structured thylakoid membranes to maintain higher rates of photosynthesis than HanFengB.

### Cooperative transcription regulatory networks in response to drought stress

**Figure 6:**
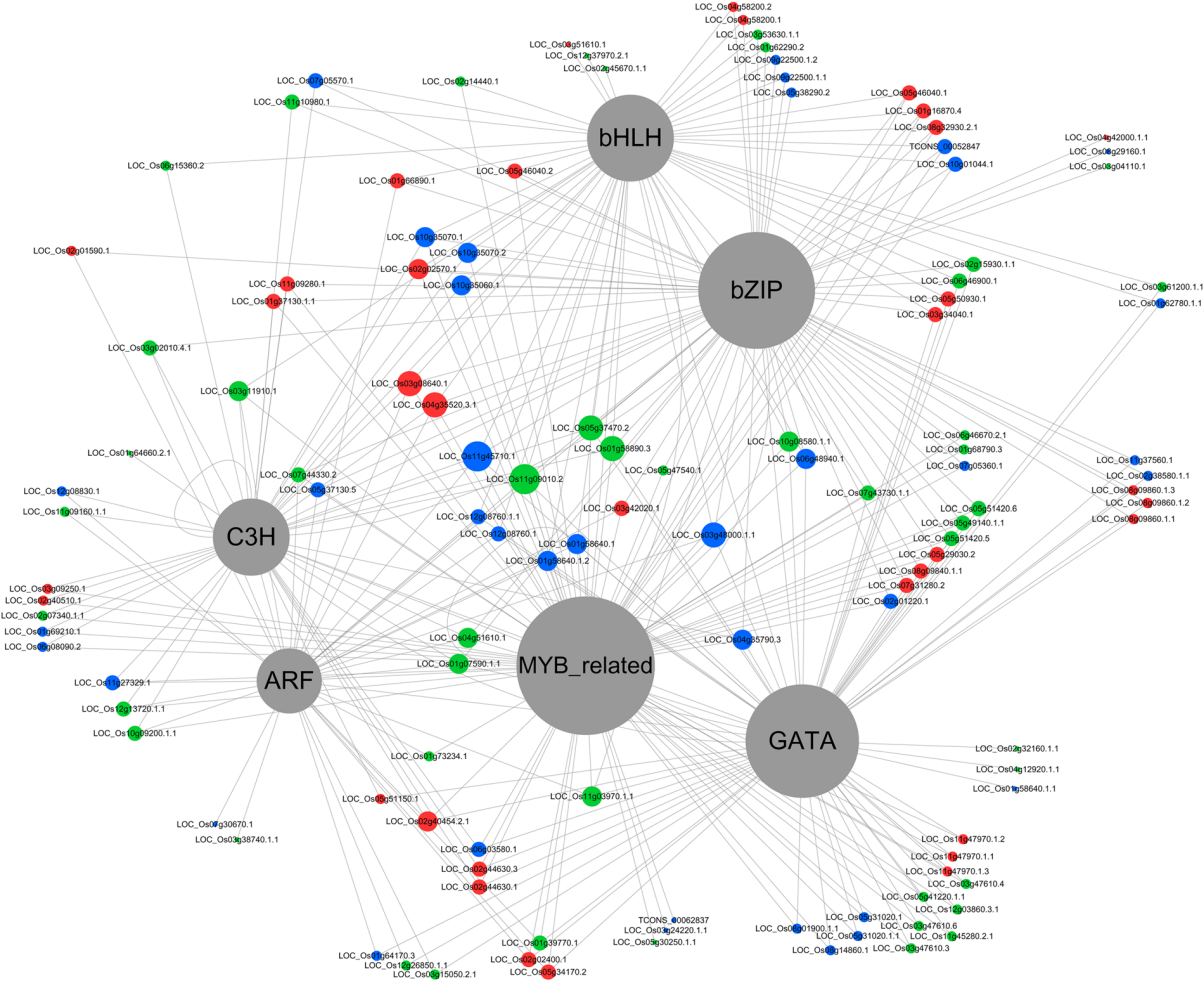
Transcription regulatory network representing the transcription factors (TFs) involved in putative TF binding sites (TFBSs) in the promoters of 135 DETs in HuHan2B. Six TF families are colored gray and are considered transcriptional activators or repressors to regulate downstream gene expression.

It has been reported that almost all of the cell activities are under the control of gene networks^27^. Notably, splicing isoforms in DETs were mainly enriched in three functional categories in HuHan2B based on GO enrichment analysis, reflecting the prevalent biological processes. To further evaluate the cooperative relationship between transcription regulation, response to abiotic stimuli and chloroplast components, we identified transcription factor families and transcription factor binding sites (TFBS) within 3 kb upstream of the transcription start sites (TSSs) of DETs with AS events in HuHan2B. We observed 15 transcripts as upstream TFs, as predicted by the Plant Transcription Factor Database (PlantTFDB v4.0). As predictions of gene regulatory interactions were generated, 6 TF families, along with ARF, bzip, bHLH, C3H, GATA and MYB-related TFs, were involved intranscriptional regulatory networks, connecting TFs with 128 putative target transcripts (Fig. 6).

Interestingly, in comparing transcriptional regulatory networks with our earlier studies^26^, this network represented biological processes that were mediated by more complex interactions between TFs and splicing isoforms and exhibited a comparable level of complexity in the interconnections between transcript functions. To further illustrate the principles of regulation, we divided TFBS nodes into three categories (Fig. 6): response to abiotic stimuli (nodes in pink), chloroplast components (nodes in blue), and TFs in both categories (nodes in green). The distribution of these categories showed that a third of TFBS transcripts in the regulatory network acted as not only cellular responses to abiotic stimuli but also as chloroplast components. More details, such as the nodes and the relationships between them, can then be layered on top of this basic network. In addition to TFs that had highly similar categorizations as functionally similar target transcripts, a combination of the activities of several TFs was observed to regulate TFBS transcripts. Numerous transcripts have more than one function according to GO annotation. In particular, distinct functional DETs had consensus cis-regulatory motifs predicted to be bound by the same TF. TFBS transcripts underwent complex regulation and indicated a mutual relationship between two biological processes by TF families. The molecular analysis supported the hypothesis that the regulation of genetic networks maintained energy metabolism in the chloroplast to enhance the drought resistance of HuHan2B, possibly due to alternative RNA splicing.

### Representative alternative splicing events of multi-exon genes in leaves during drought

**Figure 7:**
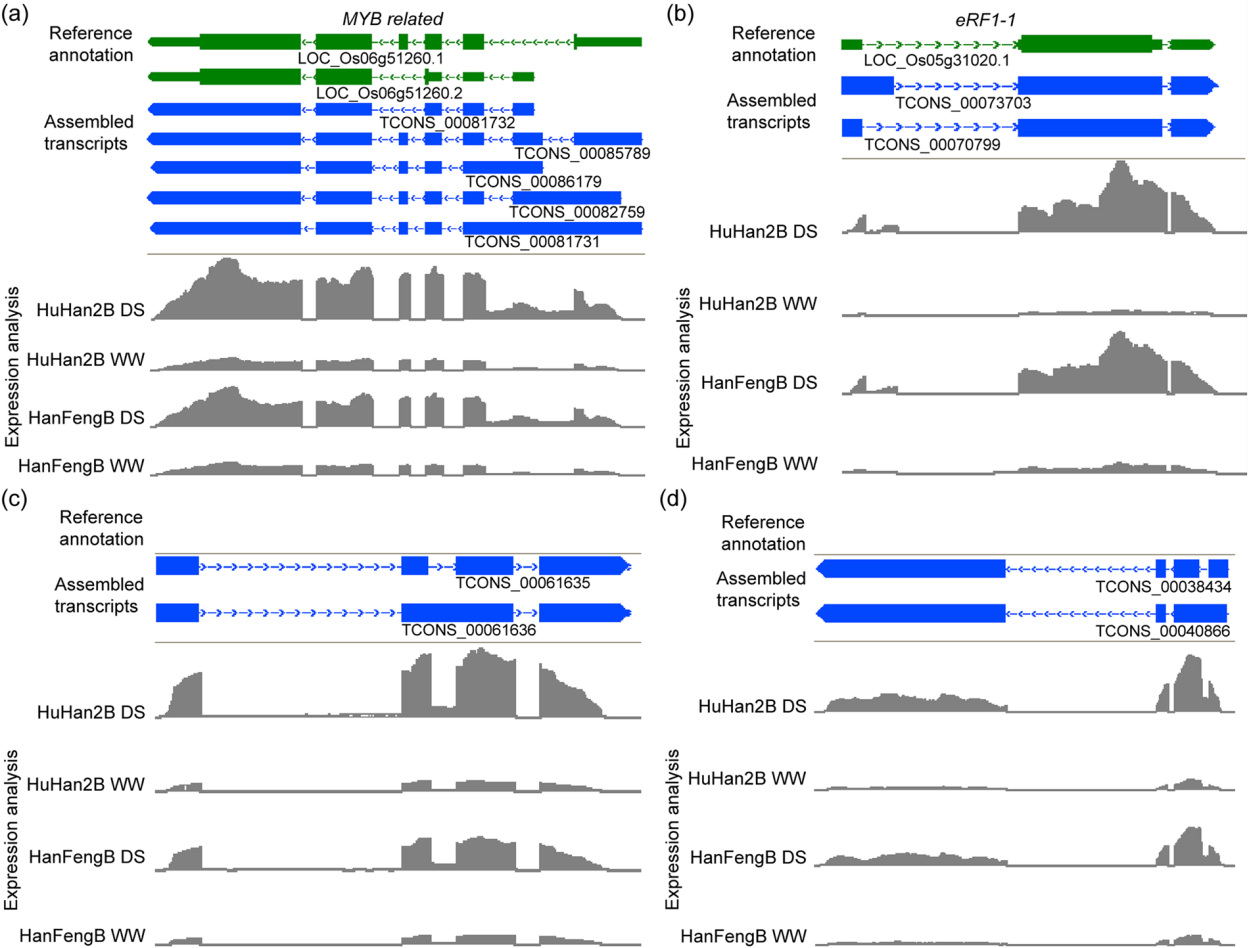
Visualization of representative transcript isoforms with AS events to determine expression changes under drought stress using the Integrative Genomics Browser (IGB). (**a**) Two exons of LOC_Os06g51260 contain alternative splicing events according to gene annotations in the reference genome. (**b**) In the first exon of LOC_Os05g31020, there is an AD event compared with the reference gene. (**c**) and (**d**) Novel transcripts are illustrated as IR events, showing higher expression under drought stress. In the IGB visualization, the exon-intron structure of each gene of the reference annotation (green) is given at the top of each panel. The gray peaks below the structure of assembled transcript (blue) indicate the RNA-Seq read density across the gene.

**Figure 8:**
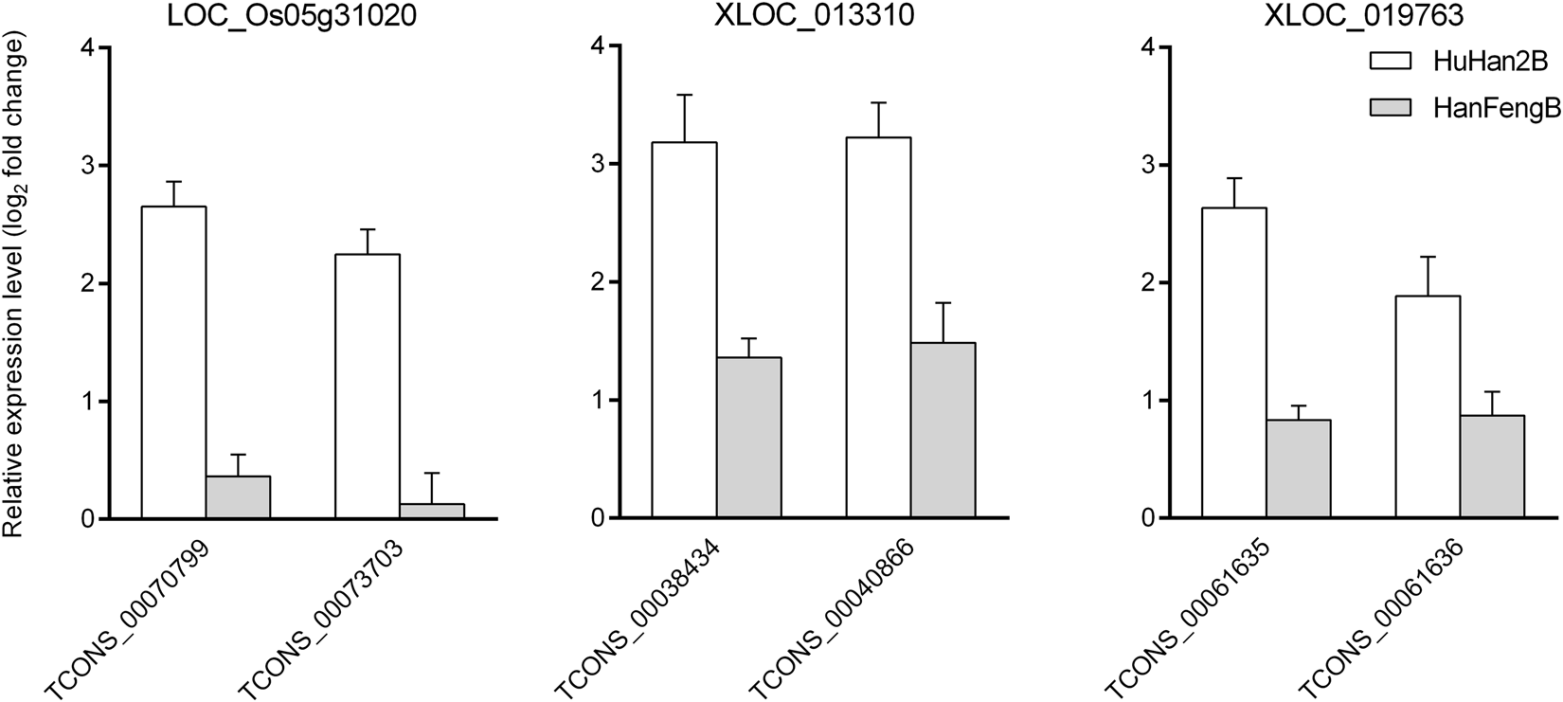
Relative expression levels of transcripts from LOC_Os05g31020, XLOC_013310, and XLOC_019763 compared with those of actin, as measured by qRT-PCR in rice seedlings at the 4-leaf stage under polyethylene glycol (PEG) treatment. The values represent the log_2_ fold changes of expression levels under drought stress and well-watered conditions.

Drought-induced splicing transcripts were the main reason for the accumulation of specific transcripts for some multi-exon genes. Four genes were visualized as examples using the Integrated Genome Browser (IGB) for differential alternative splicing events from RNA-Seq data (Fig. 7). We observed a drought-responsive diversity of transcript isoforms from each rice variety. For example, a total of five transcript isoforms (TCONS_00081731, TCONS_00081732, TCONS_00082759, TCONS_00085789, and TCONS_00086179) were identified for LOC_Os06g51260 (encoding a MYB-family transcription factor) in our transcriptome data, and their expression levels were significantly higher in both HuHan2B and HanFengB under drought stress than under well-watered conditions (Fig. 7a). Two transcript isoforms (TCONS_00073703 and TCONS_00070799) for LOC_Os05g31020 (eukaryotic release factor 1) showed enhanced expression in both rice varieties under drought stress (Fig. 7b). In addition, various novel transcripts, such as TCONS_00061635, TCONS_00061636, TCONS_00038340 and TCONS_00040866, were significantly up-regulated in both HuHan2B and HanFengB under drought stress and were expressed at higher levels in HuHan2B under drought stress than in HanFengB and under well-watered conditions (Fig. 7c and d). Some AS events were validated by qRT-PCR using intron-flanking primers. The amount of the corresponding PCR products was increased under drought stress, consistent with the RNA-Seq data (Fig. 8).

## Discussion

Over the years, suitable rice cultivars, mainly WDR, have been bred to withstand the effects of drought^3,26,28,29^. We have shown that conventional selection in water-limited environments can integrate multiple genes related to drought resistance into the same genetic network^26^. However, alternatively spliced mRNAs might modify phenotypic variation under abiotic and biotic stresses and affect overall drought resistance and growth^21,23,30^. Therefore, in this study, we addressed the impact of drought stress on AS and transcriptome profiles in offspring HuHan2B and its breeding parent HanFengB. Results from reference-based transcriptome assembly generated similar transcript isoforms (Figs 1, 2 and 3) and confirmed the limited genetic variation between the two varieties. Our results provided further evidence for genetic improvement of drought resistance in WDR breeding, possibly due to alterations in isoform-level mRNA abundances and AS patterns in multi-exon genes.

RNA AS is an important mechanism for alteration of the transcriptome to enhance drought resistance in breeding offspring. Our results suggested that at least 30% of intron-containing genes are alternatively spliced in rice leaves under drought stress, consistent with previous studies^21,31,32^. In our data, six known types of AS models were investigated, and IR was the most prevalent AS type in both rice varieties. Moreover, the ratio of IR events (approximately 41% of AS events) in the assembled transcripts was less frequent than in *Arabidopsis* and *Zea mays* ^16,30^ and similar to that in cotton and sorghum bicolor^18,32^. A greater percentage (50%) of DETs was involved in AS events (Fig. 4). We found that the proportion of shared DETs with AS events between HuHan2B and HanFengB was smaller. Furthermore, functional categories DETs with AS events were differentially over-represented in each rice variety. These differences suggested that AS isoforms might have experienced artificial selective pressure during breeding. DETs with AS events from biological processes, response to abiotic stimuli and chloroplast components were all predicted to be regulated by six TF families. Notably, the effects of drought stress on photosynthesis are directly limited by damage to the photosynthetic apparatus of chloroplasts and potential stress responses^10^. The regulatory network of TFs showed cooperation of two subnetworks, response to abiotic stimuli and chloroplast components, to maintain the energy metabolism of the chloroplast to enhance the drought resistance of HuHan2B under drought stress^33^. These findings indicate that AS has contributed to the drought stress response, with transcriptional changes having pivotal regulatory functions.

The presence of transcription factors in drought-responsive genes with AS events adds a new level of complexity to the regulation of gene expression in plants^13^. Splicing and regulatory activities can explain the links between allelic variation and regulatory mechanisms in the breeding offspring HuHan2B. As previously mentioned in the genetic determination of the enhanced drought resistance of HuHan2B, the major-effect genes were congregated and transformed into the offspring by breeding, and the inherited allele-related differentially expressed gene were enriched in the regulatory network^26^. In fact, as DETs with AS events were studied, we found that there was no co-occurrence of single-nucleotide polymorphisms (SNPs) and DETs with IR events using SNPlice tools^34^. Further study is needed to uncover the relationships between allelic variation and AS and genetic determination. However, the distinct splicing isoforms of TF are generated well after transcription to respond quickly to changing environmental conditions^35^. Considering that the MYB family could bind to approximately 60% of the total TFBS transcripts in the transcriptional regulatory network in HuHan2B under drought stress, LOC_Os06g51260 (*MYB related*) may play a significant role in drought resistance. MYB, together with other transcription factors, including ARF, bzip, bHLH, C3H and GATA, are key regulators in the plant response to drought^36^. These TFs may help promote rapid regulation of cellular subnetworks, such as response to abiotic stimuli and chloroplast components, to supply adequate energy required in the response to drought stress^37,38^. In our previous study, we found that LOC_Os05g31020 (*eRF1-1*), with allelic variation, was up-regulated in a TF regulatory network and generated two splicing transcripts. Both TCONS_00070799 and TCONS_00073703 were significantly up-regulated in RNA-Seq data and were also validated by qRT-PCR. Two other novel TFBS transcripts were more abundant in HuHan2B than in HanFengB (Fig. 8). In DETs with AS events, parts of drought-responsive genes in our previous study were involved in the transcriptional regulatory network of TFs and TFBS transcripts, such as the multi-exon genes LOC_Os01g04590 and LOC_Os001g07280^26^. As has been extensively studied in drought resistance in plants, enhanced cooperative regulation by cross-talk could be due to AS having a role in the alternative expression of transcripts from multi-exon genes.

Alternative splicing was a prominent feature resulting from different conditions in rice leaves. Multiple mature mRNAs were generated from a multi-exon gene by AS, where different combinations of splice sites were used. Additionally, alternative RNA splicing resulted in changes to the protein coding sequence, with a possible loss or gain of function. Introns had important roles in the RNA posttranscriptional modifications to diversify protein sequences by various AS events. In addition, their expression levels correlated greatly with gene expression. Our study found that most AS isoforms were accumulated or reduced in cooperation with the up- or down-regulation of known transcript expression profiles. In particular, under drought stress conditions, AS was an important mechanism controlling the different physiological properties of DETs in HuHan2B and HanFengB. Our results showed a small proportion of common DETs (~20%) between the two cultivars. The functional role of AS was also reflected in the specifically enriched GO classifications of DETs with AS events. Although the generation mechanism of AS is not clear, their features are universal in eukaryotes.

In summary, our study provided an extensive overview of the transcriptome diversity of the offspring HuHan2B and the recurrent parent HanFengB and highlighted that regulation of AS was critical to respond to changes in the environment related to drought. The identified novel transcripts and transcript isoforms were supplementary to differentially expressed genes and reflected the role in response to drought stress. The transcription regulatory network could be divided into two subnetworks based on cellular responses to abiotic stimuli and chloroplast components. The findings suggested that AS complexity contributed a new regulatory function to increase cooperative regulation of genetic networks through alternative expression of transcripts from a multi-exon gene. Overall, the candidate genes generated in this study can be used to perform genetic modifications to obtain high-yielding and drought-resistant rice breeds.

## Materials and Methods

### Plant materials

Two rice varieties, the offspring HuHan2B and the parent HanFengB, were collected from Shanghai Agrobiological Gene Center, China. HanFengB is a maintainer of the japonica hybrid rice combination Hanyouxiangqing that has been commercialized in Shanghai for decades. HuHan2B is a drought-resistant maintainer developed from backcross breeding using HuHan3 as the donor of drought resistance and HanFengB, susceptible to drought, as the recurrent parent. The majority of the HuHan2B genome is derived from the recurrent parent HanFengB due to successive backcross breeding^3,26^. One of the parents of HuHan3 was African upland variety IRAT109, which contributed drought resistance to HuHan2B.

### Drought stress treatments and RNA-Seq

As described in our previous study, drought stress field experiments were performed with a split randomized block design with three replicates. Presoaked seeds were sown in a nursery, and seedlings were then transplanted to the field on June 15, 2014. Irrigation was withdrawn on July 16, initiating the drought stress in one split area (blocks with drought stress treatment). When soil moisture declined from 23.1% to 10.0%, leaf sampling for RNA-Seq was performed on days 20, 25, and 37 after water withholding. All leaf tissues were harvested at 13:00–14:00, immediately frozen in liquid nitrogen and stored at −80 °C until RNA-Seq library preparation. The raw RNA-Seq data for the 12 pools from HanFengB and HuHan2B at three time points under well-watered and drought stress conditions were deposited in the NCBI Sequence Read Achieve (SRA) with the BioProject number PRJNA260762 (under experiments SRX1521275 to SRX1521286).

### Reference-based transcriptome assembly

To improve the accuracy of the transcriptome assembly, super-reads were first assembled de novo from original RNA-Seq data using the MaSuRCA (v3.1.3) genome assembler^39^. The extended reads were converted into longer contigs as super-reads with an average length of 210 bp. The assembled super-reads and the two unassembled paired reads files were then mapped to the Nipponbare reference genome (MSU 7.0) using TopHat2 (v2.0.13) with several modifications to the default parameters^40^: –min-intron-length 50 and –max-intron-length 50,000. According to the size of most known introns in Eukaryotes^41^, the intron lengths were expected to range between 50 bp and 50,000 bp. HuHan2B and HanFengB transcriptomes from each library were reconstructed using StringTie (v1.2.3) with the default parameters^42^ and using a reference annotation file (in GFF3 format from MSU 7.0) to guide the assembly process. Consensus assembly transcripts were generated and compared with the annotated Nipponbare reference genome via gffcompare. According to class code annotation, transcripts were categorized into known isoforms, novel isoforms, and novel intergenic transcripts. Primary transcript filtering was performed by accepting transcript expression above a threshold of FPKM 0.1 in at least three libraries and FPKM >= 1 in at least one library. A high-confidence set of transcripts was generated for the subsequent analysis. A 4-way Venn diagram web tool was used to generate figures for overlapping DETs^43^.

### Functional annotation of the assembled transcripts

All assembled transcripts were searched against several public protein databases, including GenBank NR (NR)^44^ and UniProtKB/Swiss-Prot (Swiss-Prot)^45^ databases, using BLASTX. The cut-off E-value was set to less than 1.0 × 10^−5^ for identifying proteins with high sequence similarity to retrieve their functional annotations. The Blast2GO program was used to retrieve functional annotations of (novel) transcripts from the GO database in the categories of biological processes, molecular function and cellular components^46^.

### AS events analysis in the assembled transcripts

We employed the Astalavista tools (version 3; http://genome.crg.es/astalavista/) with default parameters to identify variations between transcripts, and consequently the underlying AS events in total transcriptomes, and landscape files were produced from GTF files for further analysis.

### Functional enrichment of alternative splicing

GO functional enrichment analysis was conducted using the combined data obtained from MSU and the functional annotation of novel isoforms and intergenic transcripts and was performed using GOatools (http://github.com/tanghaibao/GOatools). Enrichment was considered significant when the p-value was less than 0.05.

### Isoform-level differential expression analysis with Ballgown

The Ballgown package of the Bioconductor software suite was used to perform differential expression analysis for the assembled and quantified transcripts^47^. The DETs were filtered with the criteria of fold change (log_2_FC) >1 or <−1, and a p-value <0.05, which were considered statistically significant for up- and down-regulation, respectively.

### Prediction of transcription factor binding sites

Putative transcription factors were predicted from DETs by PlantTFDB (http://planttfdb.cbi.pku.edu.cn/)^48^. Because TFs act at the DNA level by binding to cis-regulatory elements of genes, we searched the PlantTFDB to identify potential TF binding sites in the promoters of DETs 3,000 bp upstream of the TSS in the Nipponbare reference genome (MSU rice genome annotation project release 7.0)^49^. The interaction networks for TF families and TFBS transcripts were visualized using an interaction graph produced in Cytoscape^50^.

### Quantitative RT-PCR validation

Quantitative real-time reverse transcription-PCR (qRT-PCR) was used to validate the reproducibility of transcriptomic expression data. Seedlings at the 4-leaf stage were treated with 20% (w/v) polyethylene glycol 6000 (PEG6000). Leaves were harvested after 6 h of treatment, frozen in liquid nitrogen and stored at −80 °C until use. All primers were designed using Primer3 (http://frodo.wi.mit.edu/) and were synthesized by Sangon Biotech. qRT-PCR was performed using a CFX96™ real-time PCR detection system (BioRad, CA, USA) following the manufacturer’s instructions. The validation was assessed with three biological replicates for each sample. The rice actin gene was selected as an internal control for normalization. Fold change was evaluated using the standard 2^−ΔΔCT^ method. This methodology produced a set of 3 genes with 6 transcripts for verification by qRT-PCR.

## Electronic supplementary material

Table S1

Table S2
